# Reply to comment on 'D-dimer and high-sensitivity C-reactive protein levels to predict venous thromboembolism recurrence after discontinuation of anticoagulation for cancer-associated thrombosis'

**DOI:** 10.1038/s41416-018-0362-9

**Published:** 2019-01-30

**Authors:** Luis Jara-Palomares, Maria Isabel Asensio-Cruz, Teresa Elias-Hernandez, Samira Marin-Romero, Remedios Otero-Candelera

**Affiliations:** 1 Medical Surgical Unit of Respiratory Diseases, Virgen del Rocio Hospital, Seville, Spain; 2 Centro de Investigación Biomédica en Red de Enfermedades Respiratorias (CIBERES), Instituto de Salud Carlos III, Madrid, Spain

**Keywords:** Biomarkers, Oncology

We appreciate the comment of Dr. Klok and colleagues on our recent publication about D-dimer and high-sensitivity C-reactive protein (CRP) levels to predict venous thromboembolism recurrence after discontinuation of anticoagulation for cancer-associated thrombosis (CAT).^1^ In our study, these biomarkers predicted VTE recurrence among patients with CAT, at 21 days after stopping anticoagulation treatment.

Klok et al. suggest another hypothesis about the role of these biomarkers, indicating that they could be markers of active cancer itself. I fully agree with them and there are several studies guiding on this way. Dirix et al. found that the D-dimer level was a clinically important marker for cancer progression, and points towards a relation between haemostasis and tumour progression.^2^ Allin et al., in an epidemiological study, found that among individuals diagnosed with cancer, those with a high baseline CRP had an 80% greater risk of early death compared with those with low CRP levels.^3^ These biomarkers are non-specific and can be increased in a lot of conditions, and future studies should confirm the utility in CAT to confirm our findings.

To stop anticoagulant treatment is a dilemma in CAT because, although there are no trials on this context, guidelines indicate continuation of anticoagulation beyond 3–6 months.^4^ For that reason, when we planned this study, we considered several conditions for stopping anticoagulant treatment. One of the exclusion criteria was a subjective assessment of the treating physician ('absence of circumstances favouring treatment maintenance based on the clinician’s discretion'). This condition was crucial, because the final decision of the clinician depends on several factors and sometimes it is impossible to include all of them as criteria or form them into a score. This subjectivity was considered in other criteria, for example, in Wells score or Hestia criteria.^5,6^

In our study, performance status and metastases were not predictors of recurrent VTE. Moreover, metastasis was included in competing risk analysis as a predefined variable and was not significant (sub-hazard ratio: 2.08; 95% CI: 0.64–6.79; *p*: 0.23).^1^ We did not observe a relationship between ECOG performance status and VTE recurrences, although 94% of patients included (*n* = 107) had ECOG performance status ≤1 and only 6% had ≥2. About oncological treatment at inclusion, they were receiving hormonal therapy (12%), 5-fluoruracil (4.5%), gemcitabine (4%), cetuximab (1.8%), bevacizumab (1%) and pemetrexed (1%), and 76% were not receiving any oncological treatment at inclusion.

It was observed by van der Hulle et al. that cured cancer patients could safely stop anticoagulants with a low risk of recurrent VTE.^7^ The problem is that active cancers implies a very different condition, and most cancers need several years of follow-up to be considered cured by an oncologist. And, in breast cancer or prostate cancer for example, some patients will receive hormonal therapy for months or years. The idea in our work was to contribute a simple biomarker to help clinicians safely stop anticoagulant treatment in CAT, and in our daily practice, all of us have patients with this condition that could obtain benefit from this approach.
